# LuminaOSL: Development and Experimental Validation of a Modular Automated Platform for Optically Stimulated Luminescence Measurements

**DOI:** 10.3390/s26185910

**Published:** 2026-09-18

**Authors:** Eren Cihan Karsu Asal, Mehmet Taştan, Hayrettin Gökozan

**Affiliations:** 1 Department of Electric, Manisa Celal Bayar University, Manisa 45030, Turkey; hayrettin.gokozan@cbu.edu.tr; 2 Department of Electronics and Automation, Manisa Celal Bayar University, Manisa 45030, Turkey; mehmet.tastan@cbu.edu.tr

**Keywords:** OSL reader, BeO, CW-OSL, LM-OSL, photon counting, smart healthcare engineering

## Abstract

Optically stimulated luminescence (OSL) is widely used in radiation dosimetry and luminescence research, creating a continuing need for flexible measurement systems capable of supporting diverse experimental protocols. In this study, LuminaOSL was designed and developed as a modular, automated, computer-controlled research platform for implementing different OSL measurement protocols. The platform integrates programmable LED-based optical stimulation, photomultiplier tube (PMT)-based photon counting, an automated eight-position sample mechanism, microcontroller-based control, and a Python-based user interface within a unified architecture. System performance was evaluated through repeated measurements using beryllium oxide (BeO), low-signal control tests, and CW-OSL and LM-OSL experiments. A cycle-to-cycle coefficient of variation of 0.41% was obtained over successive measurement cycles, with no missing data observed during data acquisition and transfer. BeO measurements yielded monotonically decreasing CW-OSL responses under constant optical stimulation and peak-shaped LM-OSL responses under linearly increasing optical stimulation. These findings indicate that LuminaOSL can support stable and reproducible measurements through the integration of programmable optical stimulation, automated sample handling, and time-controlled photon counting, with potential applicability to radiation dosimetry and smart healthcare engineering research.

## 1. Introduction

OSL is a measurement technique based on the storage of charge carriers, generated in insulating or wide-band-gap solid materials exposed to ionizing radiation, at trapping levels within the crystal structure, followed by their release through optical stimulation at an appropriate wavelength [1]. The luminescence emitted when the released charge carriers recombine at recombination centers provides information on the radiation history and trapping structure of the material. Owing to these characteristics, OSL has been widely employed in a range of applications, including personal and medical dosimetry [2], environmental radiation monitoring [3], retrospective dosimetry, archaeological and geological dating, and the characterization of luminescent materials [4,5]. The reliability of OSL measurements depends not only on the sensitivity of the dosimetric material but also on the stable and coordinated operation of the optical stimulation, luminescence detection, sample positioning, electronic control, and data acquisition components. Controlled application of the stimulation-light intensity and temporal profile, effective separation of the intense stimulation light from the weak luminescence signal, reliable detection of low-level photons, and reproducible positioning of the sample within the same optical geometry are fundamental requirements for such systems. In addition, particularly for time-resolved OSL measurements, maintaining accurate acquisition timing and limiting the influence of delays arising from computer communication or user-interface operations are important considerations [6]. OSL readers have been developed with different stimulation, detection, sample-handling, and data acquisition architectures depending on their intended application. Commercial laboratory readers provide a high degree of standardization and automation, whereas research-oriented platforms offer greater flexibility through programmable stimulation conditions, reconfigurable experimental sequences, and hardware–software architectures that can be adapted to specific research requirements. In addition to photomultiplier tube (PMT)-based laboratory readers, microcontroller-controlled systems and research OSL platforms incorporating automated sample management have been reported in the literature [7,8,9,10]. Studies on automated OSL readers have further demonstrated that optical stimulation, photon detection, sample movement, and computer-based control can be integrated within a unified research system [11,12,13,14]. The temporal form of an OSL signal is directly governed by the applied optical stimulation protocol. In CW-OSL, the stimulation intensity remains constant throughout the measurement, whereas in LM-OSL it is increased linearly with time [15,16]. The application of this approach to different luminescent materials has also highlighted the importance of programmable optical stimulation in OSL measurements.

BeO is one of the dosimetric materials of considerable interest for OSL applications. Systematic investigations in the late 1990s demonstrated an intense OSL response from irradiated BeO ceramics and characterized key features relevant to its use in radiation dosimetry [17]. Subsequent studies further developed BeO-based OSL dosimetry through dedicated reader concepts and applications, including 2D-OSL dosimetry, personal dosimetry, and the BeOmax system [18,19,20,21]. These developments, together with the near-tissue-equivalent effective atomic number and favorable luminescence characteristics of BeO, have supported its investigation particularly in personal and clinical dosimetry studies [22,23,24,25,26], areas that are increasingly relevant to the development of automated measurement technologies for smart healthcare applications [27,28].

Experimental studies on BeO have reported characteristic decaying luminescence responses under CW-OSL and peak-shaped signal profiles associated with linearly increasing optical stimulation under LM-OSL [29]. These established OSL characteristics make BeO a suitable reference material for experimentally evaluating the basic optical, mechanical, and photon-counting performance of a newly developed measurement system [30]. For a research-oriented OSL platform, the manner in which optical components are integrated within the system is as important as the selection of the components themselves. LED-based optical stimulation, PMT-based photon counting, and optical filter combinations that separate the stimulation light from the luminescence emission are commonly employed in OSL readers [31]. At the same time, effective isolation of control components such as the microcontroller, motor and LED drivers, and power electronics from the sensitive optical measurement volume is important for limiting unwanted optical contributions. Likewise, separating time-critical photon-counting operations from computer-side communication and user-interface processes provides a practical system-level approach for preserving acquisition timing during extended measurement sequences.

In this study, LuminaOSL was developed as a modular, automated, computer-controlled measurement platform for research-oriented OSL applications. The system integrates programmable optical stimulation, PMT-based photon counting, automated eight-sample transport and positioning, embedded control, and a Python (3.9.6v) based user interface within a unified architecture. The platform was designed to separate time-critical measurement operations from computer-side communication and user-interface processes while maintaining optical isolation between the measurement and electronic control compartments. Its performance was assessed through system-level validation tests and CW-OSL and LM-OSL measurements using BeO dosimeters, with particular emphasis on measurement repeatability, data acquisition integrity, and the implementation of different optical stimulation profiles.

## 2. Materials and Methods

This section describes the hardware and software architecture of the LuminaOSL platform and the methodology used for experimental validation. The principal design criteria were effective optical isolation, reproducible mechanical positioning, preservation of data integrity, and independence of measurement timing from computer-induced delays.

### 2.1. LuminaOSL System Architecture

**Figure 1:**
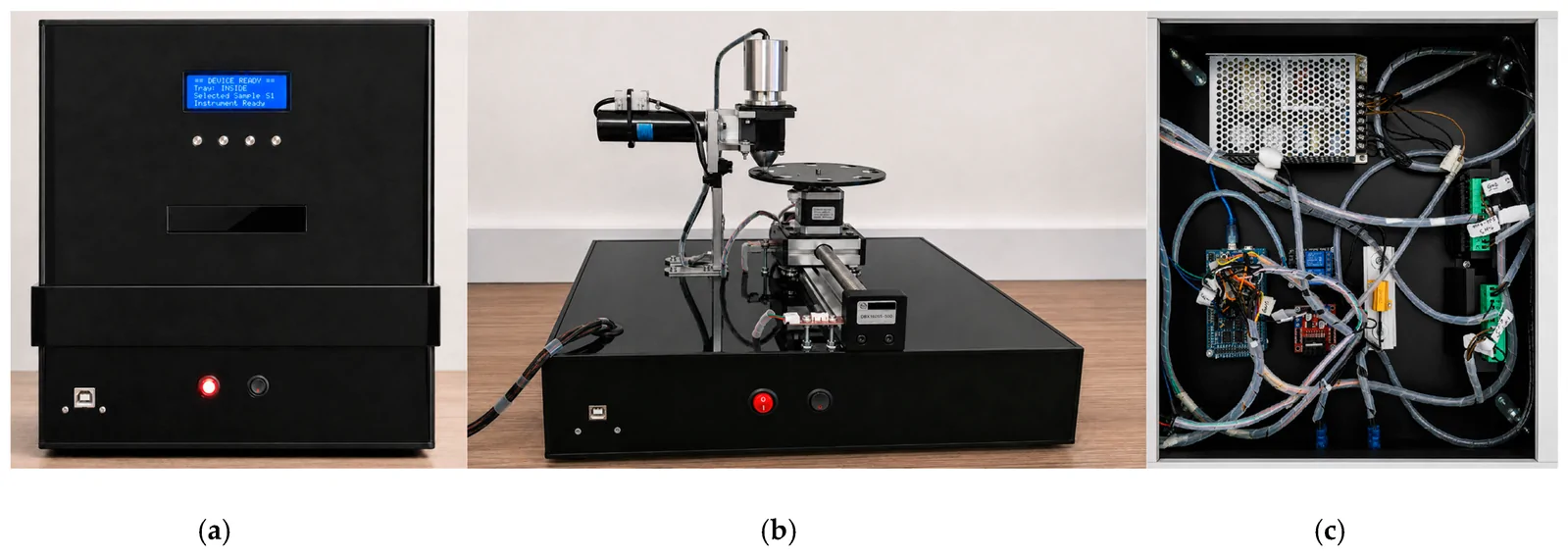
Overall physical configuration and two-compartment enclosure architecture of the LuminaOSL system. (**a**) Overall view of the system, (**b**) upper opto-mechanical compartment containing the optical measurement head and sample-positioning mechanism, and (**c**) lower compartment housing the control electronics, motor drivers, and power units.

LuminaOSL was designed as a modular and extensible research platform that enables different OSL measurements to be performed using a common hardware and software infrastructure. The system comprises four integrated subsystems: the optical stimulation and detection unit, the automated sample-transport and positioning mechanism, the electronic control system, and the computer-based data acquisition software. This architecture allows individual subsystems to be independently modified or upgraded to meet different experimental requirements while preserving the overall measurement framework. Research-oriented OSL readers reported in the literature commonly employ optical stimulation, optical filtering, photodetection, automated sample handling, and computer-based control within different system architectures depending on the intended application [11,12,13,14,32]. LuminaOSL is housed in a light-tight enclosure measuring 40 × 40 × 40 cm and constructed from 5 mm thick opaque black acrylic. The enclosure is divided into two functional compartments: an upper 30 × 40 × 40 cm opto-mechanical measurement compartment and a lower 10 × 40 × 40 cm electronic control and power compartment. The overall physical configuration, front panel, and two-compartment internal architecture of the system are shown in Figure 1.

Figure 1a shows the front panel of the system. The LCD displays system status and relevant operating information. Below the LCD, two control buttons are used to extend and retract the sample disc, while two additional buttons are used to sequentially index the sample positions when the disc is outside the enclosure. During sample loading, the front access cover opens automatically, after which the sample disc is extended outside the enclosure. The indexing controls are then used to sequentially bring each sample position to the loading position, allowing the dosimeters to be loaded individually. After loading, the sample disc is retracted into the enclosure and the access cover closes automatically, restoring the light-tight configuration. The lower part of the front panel also includes the USB connection between the MCU and the computer, the main system power control, and an independent ON/OFF control for the PMT.

Figure 1b shows the general physical arrangement of the upper opto-mechanical measurement compartment and the sample-transport and positioning structure.

Figure 1c shows the lower electronic control and power compartment, which is optically isolated from the measurement volume. This physical separation limits unwanted light originating from the electronic and power components from reaching the optical detection region, while allowing maintenance and hardware modifications to be performed without disturbing the optical measurement compartment.

### 2.2. Opto-Mechanical Measurement System

**Figure 2:**
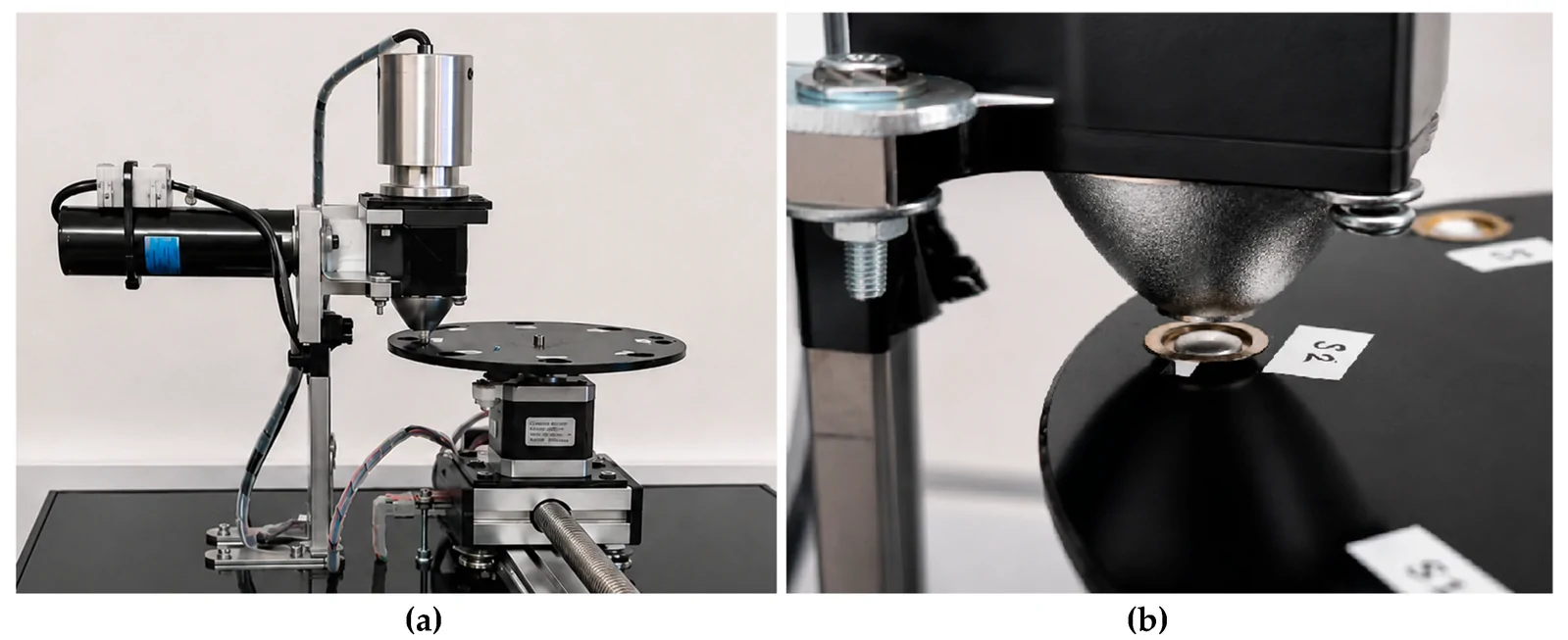
Sample-transport and positioning mechanism of the LuminaOSL system. (**a**) Overall arrangement of the linear sample tray, eight-position rotating sample disc, and optical measurement head; (**b**) positioning of the selected sample along the fixed optical measurement axis.

The opto-mechanical system of LuminaOSL was designed to integrate the transport of dosimeter samples into the light-tight measurement region, positioning of the selected sample along the optical axis, delivery of the stimulation light to the sample, and collection of the resulting luminescence into the detection channel. The system comprises a linear sample tray, an eight-position rotating sample disc, position-sensing elements, and a fixed optical measurement head. The sample-transport system incorporates two axes of motion: linear tray translation and rotary sample positioning. The linear axis moves the sample tray between a user-accessible loading position and the light-tight measurement position. A lead-screw mechanism driven by a stepper motor is employed for this motion. The travel limits of the tray are monitored by inner and outer position sensors, and measurement is initiated only after the tray reaches the predefined measurement position. The rotating sample disc contains eight sample positions arranged at equal angular intervals and is actuated by a separate stepper motor. Its angular reference is established using an optical position sensor. Upon system initialization, the disc moves to the reference position, where the Sample 1 position is identified by the optical sensor. The remaining sample positions are determined from this reference point according to the corresponding motor pulse counts. To prevent the accumulation of positioning deviations over successive operations, the system returns to the reference position before each measurement and subsequently moves the selected sample from this reference to the measurement position. The overall configuration of the sample-transport and positioning mechanism is shown in Figure 2.

**Figure 3:**
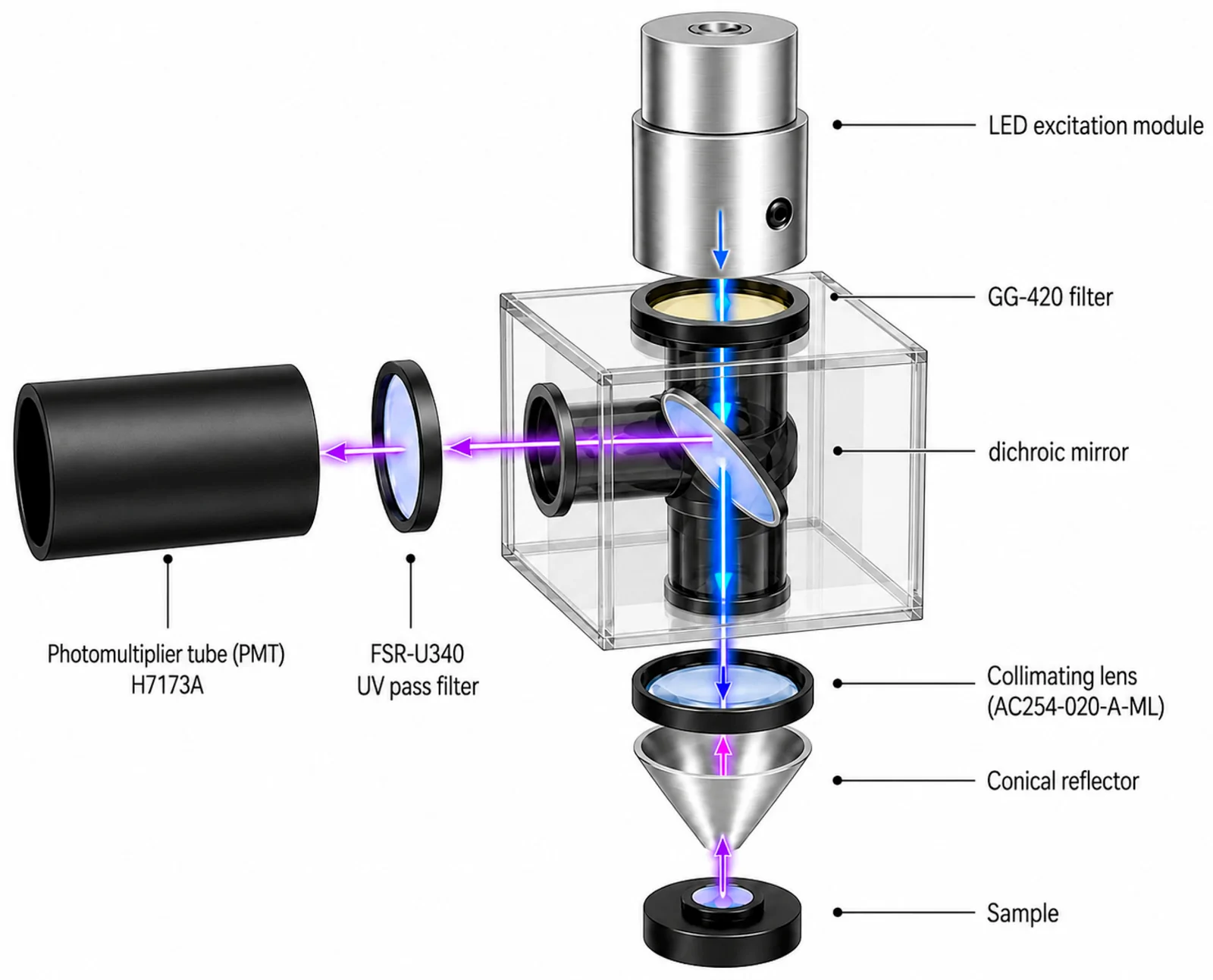
Optical stimulation and luminescence detection geometry of the LuminaOSL measurement head.

In this positioning scheme, the optical measurement head remains stationary, while sample changes are performed exclusively through movement of the transport mechanism. Consequently, each selected sample is brought to the same measurement axis without requiring realignment of the optical components between measurements. Re-establishing the reference position before each measurement and positioning the target sample relative to this reference maintains a consistent mechanical reference throughout the automated eight-sample measurement sequence. The optical measurement head was constructed within an opaque black acrylic housing with approximate dimensions of 4 × 4 × 4 cm to provide blue-light stimulation of the sample and to collect and direct the resulting The image resolution was set to 500 dpi by making the necessary adjustments.low-intensity OSL emission toward the PMT. The stimulation and detection paths were geometrically and spectrally separated to limit the amount of intense stimulation light reaching the detection channel while allowing luminescence emitted by the sample to be transmitted to the PMT. A 3W Cree blue power LED with a peak emission wavelength of approximately 475 nm was used as the stimulation source. Recent studies on BeO-based OSL systems have shown that optical stimulation power is an important experimental parameter affecting signal magnitude and signal-to-noise performance [33]. The LED has a rated drive current of 700 mA; however, in the present system, the drive current was limited to approximately 500 mA at 100% PWM to reduce thermal loading and allow stable operation without additional active cooling. The distance from the LED source to the sample position is approximately 100 mm, and the final aperture of the conical reflector through which the stimulation light reaches the sample has a diameter of 10 mm. The absolute optical irradiance at the sample position was not independently calibrated in the present study. A Schott GG-420 filter, 25 mm in diameter and 3 mm thick, is positioned at the LED output. After passing through the filter, the stimulation light is directed toward the sample by a Thorlabs dichroic mirror positioned at an angle of approximately 45° within the optical head and is delivered to the measurement region through a Thorlabs AC254-020-A-ML achromatic lens. A conical aluminum reflector located between the lens and the sample assists in directing the stimulation light toward the sample region and in coupling the luminescence emitted by the sample into the optical collection path. The luminescence emitted from the sample is directed into the detection path and passes through an FSR-U340 UV-pass filter before reaching the PMT. The combination of the GG-420 filter, dichroic mirror, and FSR-U340 filter was employed to limit the component of the blue stimulation light entering the detection channel while allowing shorter-wavelength luminescence emission to reach the PMT. A Hamamatsu H7173A photomultiplier tube module was used as the detection unit. The R3550-series PMT incorporated in the module has a spectral response range of 300–650 nm, with a peak response at 375 nm, according to the manufacturer’s specifications [34]. Photon-counting pulses generated by the detector are transmitted to the control unit, where the pulses accumulated within the defined integration interval are recorded as the photon count for the corresponding measurement point. The LED, filters, lens, dichroic mirror, reflector, and detector are mounted on fixed supports. The optical stimulation and detection geometry is shown in Figure 3.

The optical arrangement of the blue LED, GG-420 filter, dichroic mirror positioned at 45°, AC254-020-A-ML achromatic lens, conical aluminum reflector, sample position, FSR-U340 UV-pass filter, and Hamamatsu H7173A is shown. The fixed optical geometry eliminates the need for optical realignment during sample changes, since only the selected sample is moved to the predefined measurement position. The combined use of spectral filtering and physical light isolation provides the optical basis for separating the intense blue stimulation light from the lower-intensity OSL emission within the same measurement volume.

### 2.3. Electronic Control System

The electronic control system of LuminaOSL was developed to coordinate optical stimulation, photon counting, sample transport, and positioning within a common control architecture. An Arduino Due serves as the central control unit and is responsible for controlling LED stimulation, acquiring photon-counting pulses from the PMT, managing the linear and rotary motion mechanisms, monitoring the position sensors, and communicating with the computer.

The optical stimulation level is regulated through the LED driver stage using a pulse-width modulation (PWM) signal generated by the Arduino Due. During CW-OSL measurements, the stimulation level is maintained constant throughout the measurement, whereas in LM-OSL measurements, the PWM duty cycle is increased linearly with time between predefined initial and final values. Different optical stimulation profiles can therefore be implemented under software control using the same electronic control architecture.

Photon-counting pulses from the PMT are processed directly by the microcontroller. The total number of pulses accumulated within each predefined integration interval is stored in memory as a single measurement point, and this process continues until the specified measurement sequence is completed. In this study, the data acquisition interval (integration time) was set to 0.5 s, with pulses from the PMT output accumulated by a hardware counter during each integration interval.

Controlling both photon counting and LED stimulation through the same control unit enables coordinated implementation of the optical stimulation profile and data acquisition timing. The mechanical motion system is also controlled by the Arduino Due.

**Figure 4:**
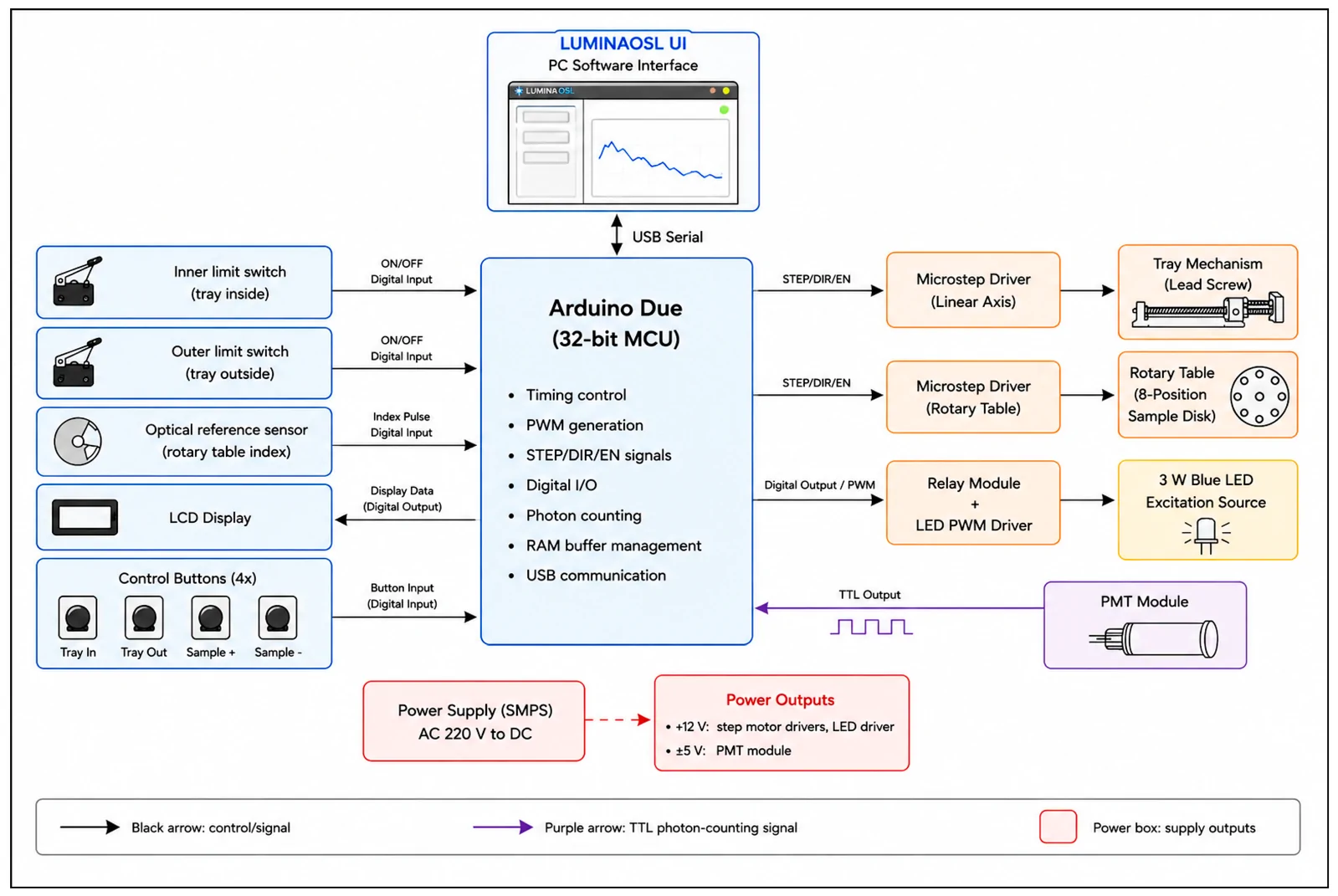
Microcontroller-based electronic control architecture of the LuminaOSL platform.

Movement of the linear tray, referencing of the rotating sample disc, and positioning of the selected sample at the measurement location are performed as part of the embedded control sequence. Mechanical status is verified using signals from the position sensors, and optical measurement is initiated only after the sample has reached the predefined measurement position. Mechanical positioning, optical stimulation, and photon counting are thus executed as sequential stages of the same automated measurement sequence rather than as independent operations. The basic architecture of the electronic control system is shown in Figure 4.

The main connections between the Arduino Due-centered control architecture and the PMT photon-counting unit, LED driver, linear and rotary motion units, position-sensing elements, local user controls, and power distribution units are shown. The centralized control architecture summarized in Figure 4 enables the optical, mechanical, and data acquisition functions involved in the measurement process to be coordinated through a single embedded system. The system is powered by a switched-mode power supply (SMPS) providing +12 V and ±5 V outputs. The +12 V output supplies the motor and LED driver stages and is also applied to the power input of the Arduino Due, whereas the ±5 V outputs supply the H7173A. The power distribution and driver electronics are located in the lower electronic compartment, physically separated from the optical measurement components. The electronic control and power infrastructure is therefore arranged in accordance with the two-compartment physical architecture of the system.

### 2.4. Software and Data Acquisition System

**Figure 5:**
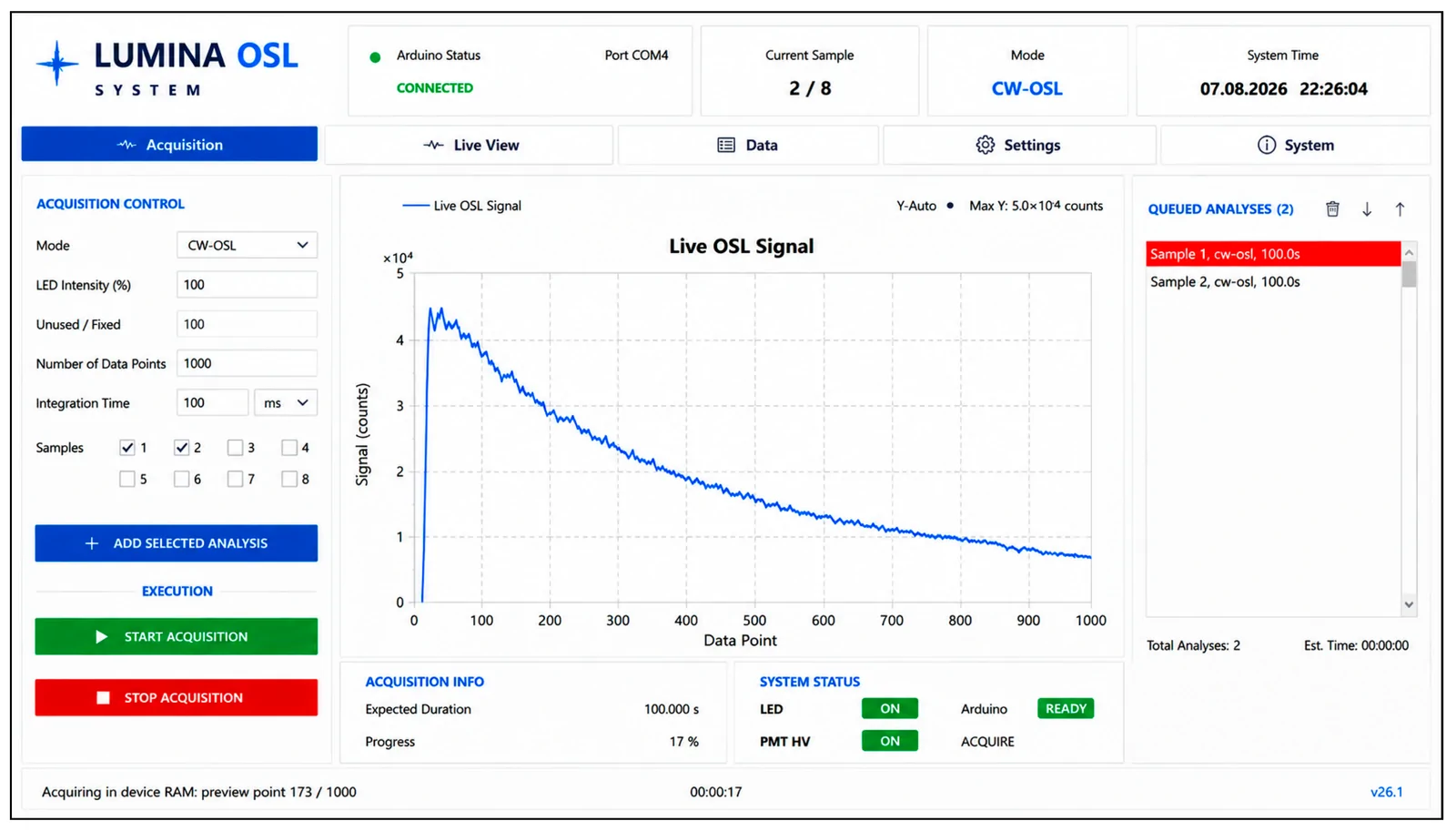
User interface of the LuminaOSL computer software.

The LuminaOSL software was developed as a Python-based user interface for defining experimental parameters, controlling the measurement process, monitoring system status, and displaying and storing the acquired data. Communication between the computer software and the Arduino Due is established via a USB serial connection. Measurement setup and user interaction are handled on the computer side, whereas timing-critical stimulation, positioning, and photon-counting operations are executed by the microcontroller. The user interface allows the measurement mode, corresponding experimental parameters, and sample sequence to be configured and, when required, multiple CW-OSL and/or LM-OSL steps with independently specified parameters to be arranged in a user-defined measurement sequence. This sequential measurement capability was not employed in the present study; instead, CW-OSL and LM-OSL measurements were performed separately for system validation. System status and measurement progress can be monitored through the interface, while the acquired data can be visualized graphically and stored after completion of the measurement for subsequent analysis. The overall layout of the user interface is shown in Figure 5.

**Figure 6:**
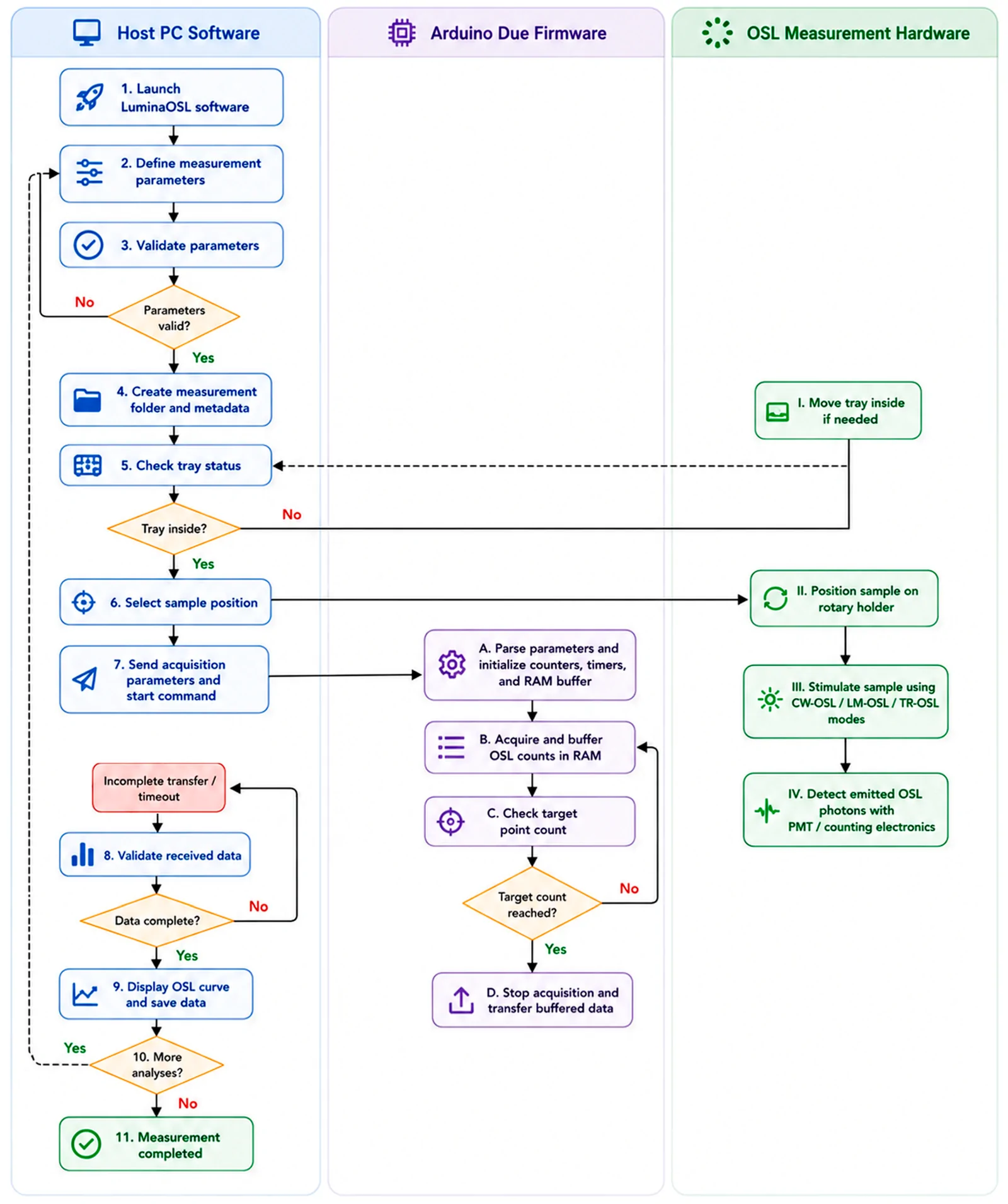
Measurement and data acquisition workflow of the LuminaOSL system.

Once the user-defined measurement parameters have been transferred to the microcontroller, the measurement sequence is executed by the embedded system. Data acquisition in LuminaOSL is based on initially storing the photon counts generated during measurement in the microcontroller memory rather than continuously transmitting them to the computer. For each integration interval, the photon count obtained from the PMT is stored in the RAM of the Arduino Due, and this process continues until the predefined measurement sequence is completed. Upon completion of the measurement, the entire dataset stored in memory is transferred to the computer in a single batch. The measurement and data acquisition workflow of the system is shown in Figure 6.

In this architecture, serial data transfer, operating-system processes, and user-interface operations do not contribute to the timing of the photon-counting sequence. The transferred data are displayed through the user interface and stored as text-based data files for subsequent analysis. This software architecture therefore separates user-defined experimental configuration from time-critical data acquisition while maintaining a unified workflow from measurement setup to data storage.

### 2.5. System Validation Procedures

Before the LuminaOSL platform was used for OSL measurements, the measurement stability and repeatability of the integrated optical, electronic, opto-mechanical, and data acquisition subsystems were evaluated through experimental validation tests. The validation procedures were designed to assess the repeatability of successive automated measurement cycles, data recording and transfer integrity, system background, and the behavior of the PMT-based photon-counting chain under low-signal conditions. An integration interval of 0.5 s was used throughout the CW-OSL and LM-OSL experiments, the LED-on and LED-off background measurements performed with an empty sample holder, and the system repeatability measurements conducted using unirradiated BeO. To evaluate the cycle-to-cycle measurement repeatability of the integrated system, 10 independent automated measurement cycles were performed under identical experimental conditions using an unirradiated BeO dosimeter. Before each measurement cycle, the system automatically returned to its reference position, after which the sample was repositioned along the fixed optical measurement axis relative to this reference. The evaluation therefore encompassed not only the temporal stability of the photon-counting chain but also the integrated cycle-to-cycle repeatability of automatic referencing, sample transport, repositioning, optical stimulation, and detection. The first two data points recorded at the beginning of each measurement sequence, before LED stimulation was activated, were excluded from the statistical evaluation. The mean photon count and within-cycle standard deviation were calculated separately for each cycle. Cycle-to-cycle repeatability was evaluated from the distribution of the mean count values obtained from the independent measurement cycles, and the coefficient of variation (CV) was calculated as the standard deviation of the cycle means divided by their overall mean and expressed as a percentage. A linear trend analysis was applied to the cycle means to assess short-term system drift.

The same measurement sequence was also used to evaluate data acquisition and transfer integrity. Photon counts were buffered in the microcontroller memory during measurement and transferred as a complete dataset to the computer software after completion of the measurement. Data integrity was assessed by comparing the number of recorded data points with the number expected for the predefined measurement sequence. This validation was performed to evaluate the data acquisition and transfer integrity of the RAM-based buffering and post-measurement batch-transfer architecture implemented in LuminaOSL. To characterize the behavior of the PMT-based photon-counting chain under low-signal conditions and to assess the system background, five independent control measurements were additionally performed using an empty sample holder under LED-off conditions and under LED-on conditions at 100% PWM duty cycle. The LED-off measurements were used to determine the baseline count level of the PMT and associated electronic counting chain in the absence of optical stimulation. The LED-on measurements enabled evaluation of the system background generated when optical stimulation was activated without a sample, as well as the stability of this background over repeated measurements. For both control conditions, the cycle means, cycle-to-cycle standard deviation, CV, and linear drift were evaluated using the same statistical approach. Following the system validation tests, the OSL measurement capability of the LuminaOSL platform was evaluated through CW-OSL and LM-OSL experiments using BeO dosimeters. Commercial BeO ceramic dosimeters (Thermalox 995, Brush Wellman Inc., Cleveland, Ohio, USA) with dimensions of Ø4 × 1 mm were used [35] and irradiated for 2, 4, 6, and 10 min using a ^90^Sr–^90^Y β-source with an activity of 650 MBq. The nominal dose rate of this source at the irradiation position was approximately 11 Gy h^−1^, based on a recent cross-calibration performed using TLD-100 (LiF: Mg, Ti) dosimeters under the same irradiation geometry [36]. Accordingly, the irradiation durations of 2, 4, 6, and 10 min correspond to nominal doses of approximately 0.37, 0.73, 1.10, and 1.83 Gy, respectively.

**Figure 7:**
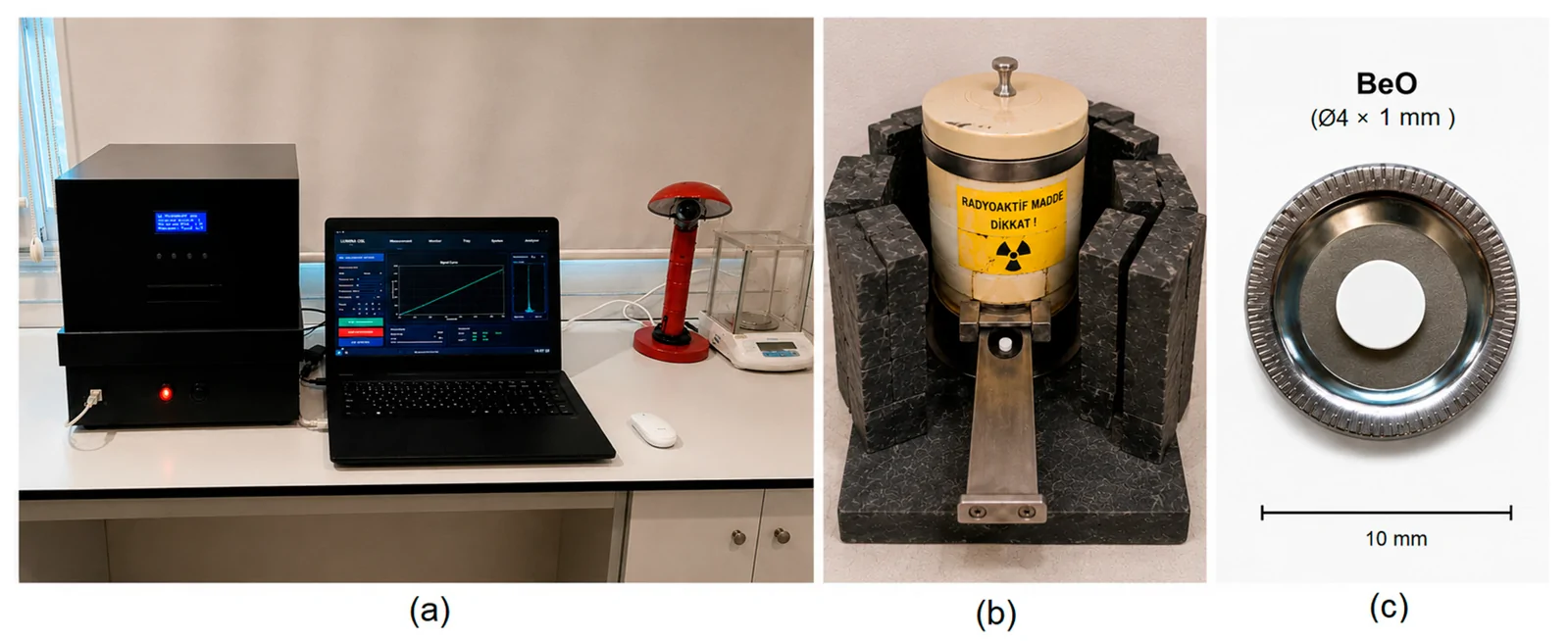
Experimental setup and materials used for OSL measurements with the LuminaOSL platform. (**a**) Laboratory arrangement of the LuminaOSL measurement system and the computer-based control/data acquisition interface, (**b**) ^90^Sr–^90^Y β-irradiation setup and sample-positioning section used for irradiation of the BeO dosimeters, and (**c**) BeO ceramic dosimeter used in the CW-OSL and LM-OSL experiments.

During CW-OSL measurements, the optical stimulation was maintained at a constant level throughout the measurement, whereas for LM-OSL measurements, the LED PWM duty cycle was increased linearly from 1% to 100%. The experimental measurement configuration of the developed LuminaOSL platform, the β-irradiation system used to irradiate the BeO dosimeters, and the BeO dosimeter employed in the experiments are shown in Figure 7.

## 3. Results and Discussion

### 3.1. System Performance and Measurement Validation

**Figure 8:**
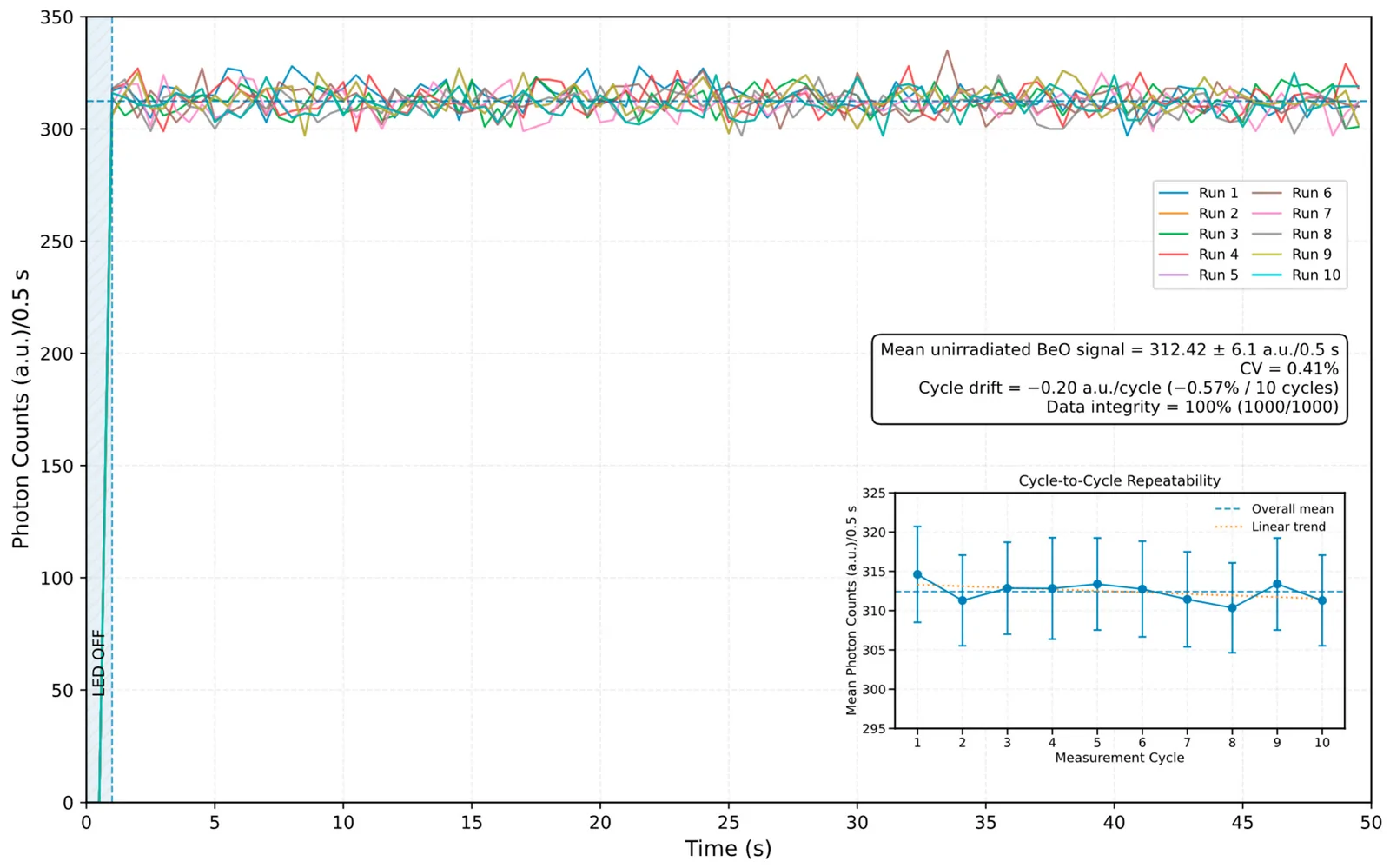
Evaluation of the integrated cycle-to-cycle measurement repeatability of the LuminaOSL platform over 10 independent automated measurement cycles using unirradiated BeO.

Before evaluating the performance of the LuminaOSL platform in OSL measurements, the stability of the measurement signal, low-level photon-counting behavior, repeatability of automated sample repositioning, and data acquisition integrity were examined. The validation tests described in Section 2.5 were conducted to assess the performance of the optical, mechanical, electronic, and data acquisition components within an integrated measurement cycle rather than to evaluate each subsystem independently. The photon-counting series obtained from 10 independent automated measurement cycles performed under identical experimental conditions using unirradiated BeO are shown in Figure 8. Because the system returned to the reference position before each measurement and the sample was subsequently repositioned at the optical measurement location relative to this reference, the experiment assessed the repeatability of the integrated system, including automatic referencing and sample positioning, rather than solely the temporal stability of the photon-counting level. Following activation of the LED stimulation, the photon-counting series remained closely grouped within a narrow range, indicating that the system maintained a stable measurement level over successive automated measurement cycles.

**Figure 9:**
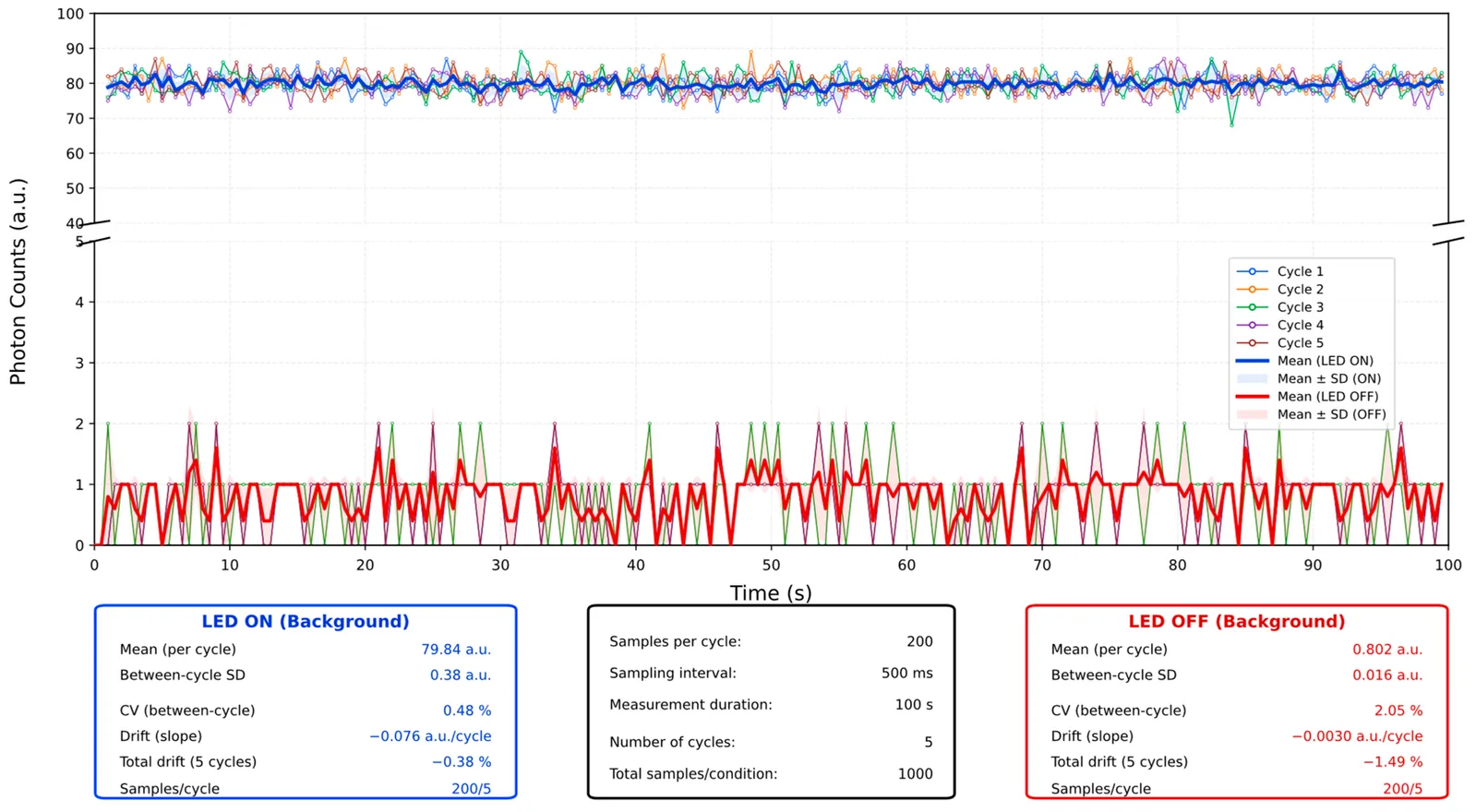
Evaluation of system background and low-level photon-counting behavior of the LuminaOSL system over five independent control measurements performed under LED-on and LED-off conditions using an empty sample holder.

The main plot shows the time-dependent photon-counting series obtained in each cycle under LED stimulation at 100% PWM duty cycle, while the inset presents the mean photon count and within-cycle standard deviation for each measurement cycle. Across the 10 cycles, a mean signal level of 312.42 ± 6.1 a.u. was obtained, with a cycle-to-cycle coefficient of variation of 0.41%. Linear trend analysis applied to the cycle means indicated a limited change of −0.20 a.u./cycle, corresponding to an overall change of approximately −0.57% across the 10 cycles. The low cycle-to-cycle variability and limited linear drift observed in Figure 8 indicate not only that the system maintained a stable photon-counting level, but also that comparable measurement conditions were reproducibly established following the referencing and automated sample-positioning procedures performed before each measurement. Accordingly, the observed variability was interpreted not as an independent positioning uncertainty associated with an individual mechanical or optical component, but as an indicator of the cycle-to-cycle measurement repeatability of the integrated system, encompassing sample transport, optical geometry, stimulation, and detection. The same experimental sequence was also used to assess data acquisition and transfer integrity. Over 10 successive measurement cycles, all 1000 expected data points were confirmed to have been recorded in the microcontroller memory and transferred completely to the computer software. This result indicates that the data acquisition architecture, in which measurement data are temporarily stored in the microcontroller memory and transferred to the computer as a complete dataset after completion of the measurement cycle, reliably preserved data integrity under the successive measurement conditions evaluated. The behavior of the PMT-based photon-counting chain under low-signal conditions and the stability of the system background were further evaluated through repeated control measurements performed under LED-on and LED-off conditions using an empty sample holder. Five independent measurement cycles were conducted for each condition, and the results are compared in Figure 9.

The LED-on measurements, performed at 100% PWM duty cycle, represent the system background under active optical stimulation, whereas the LED-off measurements represent the dark baseline count of the PMT and associated electronic counting chain. The difference in count levels between Figure 8 and Figure 9 reflects the different measurement configurations, since Figure 8 was obtained with an unirradiated BeO sample in the measurement position, whereas Figure 9 was acquired using an empty sample holder. Under the LED-on condition, the background count level derived from the cycle means was 79.84 ± 0.38 a.u., with a cycle-to-cycle coefficient of variation of 0.48%. A linear drift of −0.076 a.u./cycle, corresponding to an overall change of approximately −0.38% across the five cycles, indicates that the system background remained largely stable over repeated measurements with the LED activated. This result shows that the background contribution generated by the system under optical stimulation in the absence of a sample can be characterized with low cycle-to-cycle variability. Under the LED-off condition, the mean count level was approximately 0.8 a.u., with a cycle-to-cycle coefficient of variation of 2.05%, indicating a low dark baseline count from the PMT and associated electronic counting chain in the absence of optical stimulation. The pronounced difference between the LED-on and LED-off levels further demonstrates that the system background generated under active optical stimulation can be clearly distinguished from the detector/electronic baseline count measured under dark conditions. These findings experimentally verify the functional coordination of the optical, mechanical, electronic, and data acquisition components within the integrated measurement sequence. Repeatability of approximately 1% has been reported for repeated OSL measurements involving repositioning of BeO dosimeters within the reader [37]. Previous studies have examined the measurement stability, sensitivity variation, and dose–response characteristics of BeO dosimeters [38], while the performance of BeO-based dosimetry systems has also been evaluated in terms of sensitivity, dose response, dose-rate dependence, and fading [24]. More recent studies have assessed the performance of BeO-based OSL systems under repeated irradiation and readout conditions, with system repeatability considered a fundamental component of clinical validation [31]. Although the low cycle-to-cycle variability and limited drift obtained with LuminaOSL cannot be directly compared quantitatively with these studies because of differences in experimental protocols and evaluation methods, they are qualitatively consistent with the stable and reproducible measurement behavior reported for BeO-based OSL systems [24,31]. To place the technical architecture and measurement capabilities of the LuminaOSL platform in the context of research-oriented OSL readers reported in the literature, the principal characteristics of selected systems are compared in Table 1. The comparison considers the optical stimulation and luminescence detection units, sample management and system architecture, control and data acquisition infrastructure, supported measurement modes, evaluated dosimeters/materials, and reported key performance characteristics.

Table 1 shows that research-oriented OSL readers employ different design approaches in terms of optical stimulation sources, detection systems, sample management, control infrastructure, and supported measurement protocols. The design approach of LuminaOSL is based not on the individual novelty of any single component, but on the integration of programmable LED-based optical stimulation, PMT-based photon counting, automated eight-position sample management, re-referencing, and CW-OSL/LM-OSL measurement functions within a common control and data acquisition architecture. However, because the performance metrics reported in the literature were obtained under different stimulation conditions, detectors, optical geometries, dosimetric materials, and measurement protocols, the numerical performance values presented in Table 1 should not be interpreted as a direct comparison of performance superiority among the systems.

### 3.2. CW-OSL Measurement Validation

**Figure 10:**
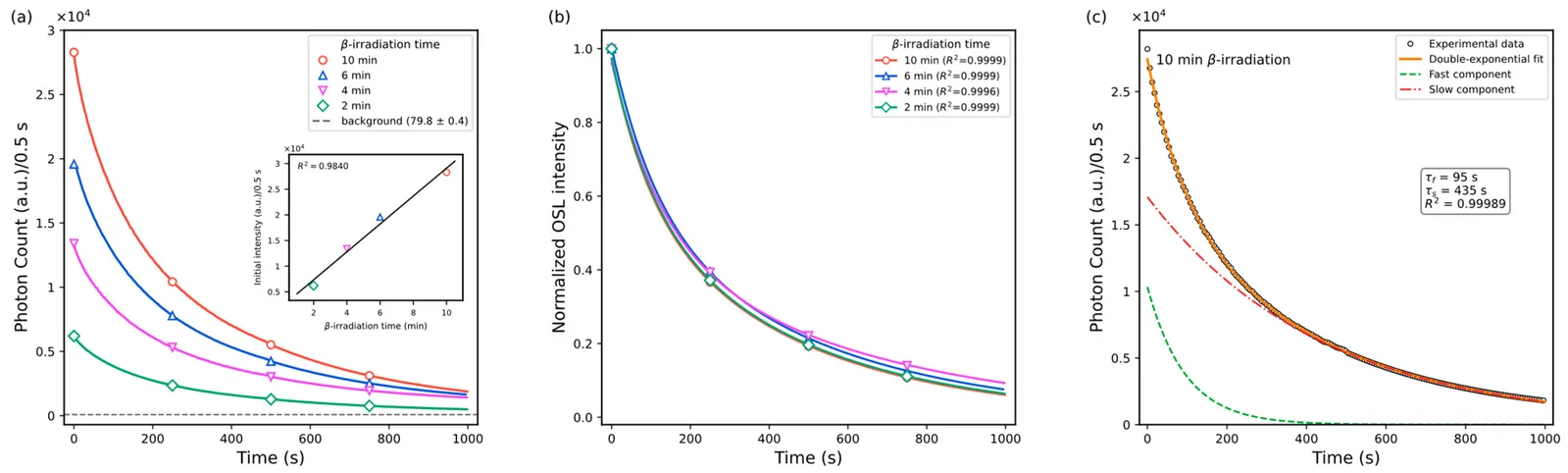
CW-OSL responses obtained from BeO dosimeters following different β-irradiation durations. (**a**) CW-OSL decay curves recorded under constant LED stimulation following β-irradiation for 2, 4, 6, and 10 min; the dashed horizontal line indicates the experimentally determined system background under the LED-on condition with an empty sample holder (79.84 ± 0.38 a.u.), while the inset shows the variation in the initial CW-OSL signal as a function of β-irradiation duration. (**b**) CW-OSL decay curves normalized to the first valid photon-count value together with the corresponding double-exponential fits. (**c**) Representative double-exponential analysis of the CW-OSL curve obtained following 10 min of β-irradiation, showing the experimental data, total fit, and fast and slow decay components.

The CW-OSL measurement capability of the LuminaOSL platform was evaluated using time-dependent luminescence responses obtained from BeO dosimeters exposed to β-irradiation for different durations. The BeO dosimeters were irradiated using a ^90^Sr–^90^Y β-source with an activity of 650 MBq. The CW-OSL measurements were initiated with minimal delay after irradiation, using the same measurement procedure throughout the experimental series. However, because measurements at controlled post-irradiation delay times were not performed, the contribution of early post-irradiation signal changes to the measured OSL response could not be quantified. Samples irradiated for 2, 4, 6, and 10 min were analyzed under identical measurement conditions. The primary objective of these experiments was not to determine the detailed trap kinetics or absolute dose response of BeO, but to establish that the developed system could record the expected CW-OSL decay behavior under constant optical stimulation and distinguish changes in signal intensity arising from different irradiation conditions. The CW-OSL responses obtained after different β-irradiation durations are presented together in Figure 10.

As shown in Figure 10a, the luminescence signal reached its maximum level at the onset of stimulation in all measurements and subsequently decreased monotonically with time. This signal profile is consistent with the expected CW-OSL behavior associated with the depletion of trapped charge carriers during constant optical stimulation. CW-OSL measurements of BeO reported in the literature have similarly exhibited decreasing, exponential-like temporal profiles following the onset of stimulation, with contributions from multiple kinetic components [41,42,43]. The observation of this characteristic time dependence in the curves recorded with LuminaOSL supports the ability of the platform to measure the expected CW-OSL response of BeO.

Increasing β-irradiation duration resulted in a systematic increase in both the initial CW-OSL signal level and the overall signal magnitude [44,45,46]. The lowest signal level was observed after 2 min of irradiation, whereas progressively higher signal levels were obtained at longer irradiation durations. As the measurements proceeded, all signal series decreased monotonically and approached the experimentally determined background region. This behavior indicates that LuminaOSL can monitor both the relatively high initial photon counts and the progressively decreasing low-level luminescence signal within the same measurement sequence. The inset in Figure 10a shows the variation in the initial CW-OSL signal as a function of β-irradiation duration. A linear fit over the investigated experimental range yielded R^2^ = 0.9840, indicating that the variation in signal level could be systematically resolved under the applied irradiation conditions. A contribution from fading to the slight deviation from linearity cannot be excluded, since increasing irradiation duration also increases the time between the generation of trapped charge during irradiation and the subsequent OSL readout. However, because controlled fading measurements were not performed, the magnitude of this contribution cannot be determined from the present data. Because only nominal source-based doses were available, the CW-OSL results were not treated as a calibrated dose–response curve.

Furthermore, because a dedicated zero-dose measurement was not included in this experiment, the origin of the non-zero intercept could not be determined from the present data, and no dosimetric significance was therefore assigned to this offset. Rather, the observed linear trend represents a system-validation result demonstrating that LuminaOSL can distinguish BeO signal levels resulting from different β-irradiation durations. This observation is qualitatively consistent with previous reports on BeO. Although an increase in CW-OSL response with increasing irradiation or dose has been demonstrated previously, those studies evaluated dosimetric response under calibrated irradiation conditions [24,41].

Accordingly, the trend observed in the present study was not compared with the literature in terms of absolute signal or dose, but only with respect to the systematic increase in the measured OSL response with increasing irradiation. For background correction, the LED-on system background was experimentally determined from separate measurements using an empty sample holder and subsequently subtracted from the CW-OSL data during data analysis. The resulting background-corrected curves were then normalized to their respective first valid values. The normalized curves are shown in Figure 10b. The broadly similar time-dependent decay profiles obtained for the different irradiation durations indicate that variations in irradiation duration were primarily reflected in the measured signal magnitude, whereas the relative CW-OSL decay profile recorded by LuminaOSL was generally preserved under the investigated conditions. The R^2^ values of the fits applied to the normalized curves ranged from 0.9996 to 0.9999, indicating close agreement between the experimental decay profiles and the double-exponential model. The limited deviations observed at later measurement times, as the signal approached the background count level, may be associated with increased relative statistical variability at low photon-count levels.

To provide a representative description of the multicomponent nature of the CW-OSL decay, the curve obtained following 10 min of β-irradiation, shown in Figure 10c, was further evaluated using a double-exponential model.

The close agreement between the experimental data and the total fitted curve indicates that the measured CW-OSL signal can be represented by components decaying on different time scales. Under CW-OSL stimulation at 100% PWM duty cycle, the fit yielded time constants of τf = 95 s and τs = 435 s for the fast and slow components, respectively. This analysis was not intended as a comprehensive component-resolved investigation of the trap kinetics of BeO, but rather as a limited validation that the CW-OSL curve recorded by LuminaOSL can be represented using a multicomponent decay model.

Overall, the CW-OSL results show that the LuminaOSL platform recorded the expected monotonic decay behavior of BeO under constant optical stimulation, distinguished changes in signal magnitude resulting from different β-irradiation durations, and continuously acquired the photon-counting sequence throughout the 1000 s measurement period. These findings experimentally validate the CW-OSL measurement function of the platform.

### 3.3. LM-OSL Measurement Validation

**Figure 11:**
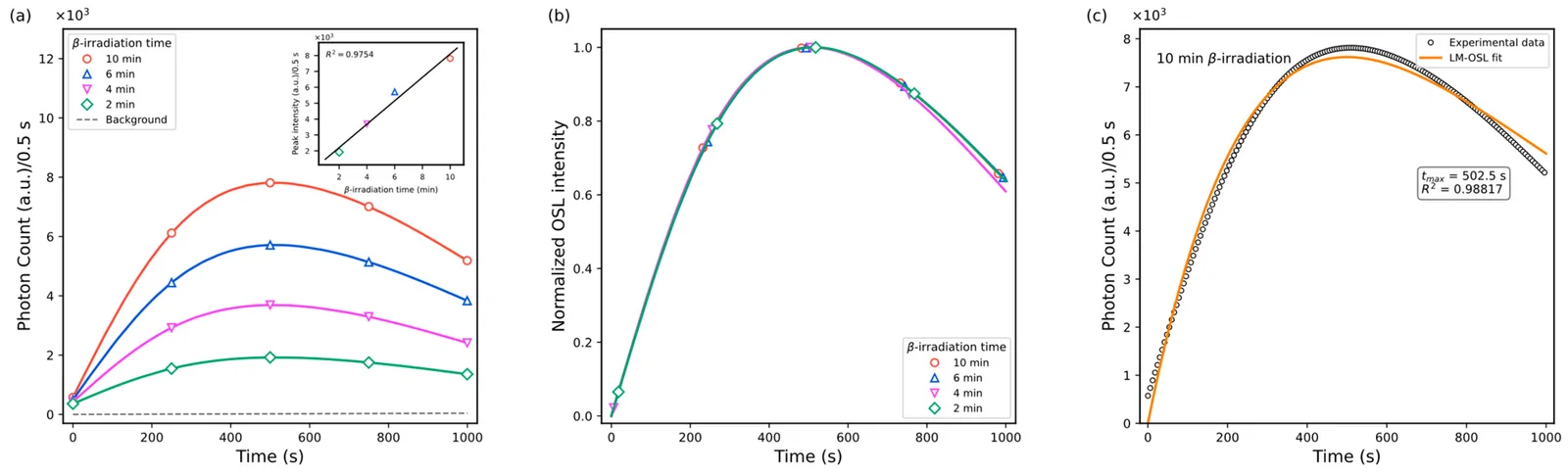
LM-OSL responses obtained from BeO dosimeters following different β-irradiation durations. (**a**) LM-OSL curves recorded after β-irradiation for 2, 4, 6, and 10 min by linearly increasing the LED PWM duty cycle from 1% to 100%; the dashed line represents the variation in background with stimulation intensity, while the inset shows the variation in LM-OSL peak intensity with β-irradiation duration together with the linear fit. (**b**) Normalized LM-OSL curves after subtraction of the initial signal level. (**c**) Representative model fit to the LM-OSL curve obtained following 10 min of β-irradiation, showing the experimental data and fitted curve.

The programmable optical stimulation capability of the LuminaOSL platform was evaluated through LM-OSL measurements performed using BeO dosimeters. For this purpose, the β-irradiation durations of 2, 4, 6, and 10 min used in the CW-OSL experiments were retained; however, instead of constant stimulation, the LED PWM duty cycle was increased linearly from 1% to 100% over the measurement period. The primary objective of these experiments was not to perform a detailed analysis of the trap structure of BeO, but to verify that the developed system could apply the defined linear stimulation profile and simultaneously record the resulting time-dependent luminescence response through photon counting. The LM-OSL results obtained after different β-irradiation durations are presented together in Figure 11.

As shown in Figure 11a, unlike the CW-OSL measurements, the LM-OSL signal initially increased, reached a distinct maximum, and subsequently decreased. Such behavior is directly consistent with the basic model established in the original description of the LM-OSL method [15]. Under linear modulation, the progressive increase in stimulation intensity produces a peak-shaped luminescence curve, in contrast to the monotonic CW-OSL decay observed under constant stimulation. Subsequent studies have similarly shown that the LM-OSL signal initially increases, passes through a maximum, and then decreases; for BeO, an approximately linear relationship between stimulation intensity and trap-emptying rate has also been reported [16]. More direct comparisons for BeO have been reported in studies investigating both CW-OSL and LM-OSL responses of BeO ceramics, including the characteristic peak structure observed under LM-OSL stimulation. It was also shown that both CW-OSL and LM-OSL curves could be approximately represented by a linear combination of two first-order components [29]. Figure 11a also shows a clear separation in signal magnitude among the curves corresponding to different β-irradiation durations. The general increase in LM-OSL signal magnitude with increasing irradiation duration indicates that the irradiation-dependent signal variation observed in the CW-OSL experiments could also be distinguished by the system under linear modulation. Previous studies on BeO have reported increasing OSL response with increasing irradiation dose and, under appropriate experimental conditions, a linear dose response [44,45,46]. Rather than serving as a dosimetric calibration, the present finding indicates that LuminaOSL can distinguish the signal levels resulting from different β-irradiation durations under the LM-OSL measurement mode. The inset in Figure 11a shows the variation in LM-OSL peak intensity as a function of β-irradiation duration. Within the investigated experimental range, the peak intensity increased systematically with irradiation duration, yielding R^2^ = 0.9754 for the linear fit. This result further supports the ability of the system to consistently distinguish LM-OSL signal levels resulting from different irradiation durations. Accordingly, dosimetric performance parameters such as the minimum detectable dose, usable dose range, and dose–response linearity were not established in the present study.

To compare the curve shapes independently of their absolute signal magnitudes, the LM-OSL data were normalized after subtraction of the initial signal level. The normalized curves are presented in Figure 11b. Following normalization, the overall peak shapes and the positions of the maxima for the different irradiation durations remained within a similar time range, indicating that the linear stimulation profile applied by the system produced comparable temporal responses across measurements despite differences in signal magnitude. For a representative evaluation of the LM-OSL temporal profile, the curve obtained following 10 min of β-irradiation, shown in Figure 11c, was further analyzed using the first-order LM-OSL model [15]. The fitting function was expressed in the peak-parameterized form as

$$I\left(t\right)=I_{m}\left(\frac{t}{t_{m}}\right)\exp\left[\frac{1}{2}\left(1-\frac{t^{2}}{t_{m}^{2}}\right)\right]$$

where *I*(*t*) is the LM-OSL intensity at time *t*, *I_m_* is the maximum LM-OSL intensity, and *t_max_* is the time at which the maximum occurs. The experimental curve reached its maximum at *t_max_* = 502.5 s, and the agreement between the experimental data and the model yielded R^2^ = 0.9881. The resulting fit indicates that the measured peak-shaped LM-OSL response can be represented by the first-order LM-OSL model. This analysis was performed not to determine detailed trap parameters or photoionization cross-sections of BeO, but to provide a quantitative representation of the LM-OSL temporal profile recorded by LuminaOSL.

Taken together, the results in Figure 11 show that LuminaOSL can perform two fundamental functions required for LM-OSL measurement within the same experimental sequence: modulation of the optical stimulation according to the defined linear profile and time-resolved recording of the corresponding photon-counting response. The qualitative agreement of the resulting peak-shaped curves with the established LM-OSL approach and with the experimental behavior reported for BeO [29] supports the operation of the system under linearly modulated stimulation. The combined evaluation of the CW-OSL and LM-OSL results provides an additional system-level validation. Using the same optical head, LED, PMT, and photon-counting infrastructure, monotonically decreasing CW-OSL curves were obtained under constant stimulation, whereas peak-shaped LM-OSL curves were observed when the stimulation was increased linearly. This distinction indicates not only that the luminescence signal was detected, but also that different optical stimulation protocols defined by the software were implemented through the electronic control system during the physical measurement process. The observation of these two distinct signal profiles using the same platform therefore provides a direct validation of its programmable measurement capability. The potential of LM-OSL peak parameters to provide information on decay characteristics was established in the original description of the method [15], while subsequent studies demonstrated the analysis of multiple LM-OSL components [16].

## 4. Conclusions

In this study, LuminaOSL, a modular, programmable, and automated measurement platform for research-oriented OSL applications, was developed and experimentally validated. A cycle-to-cycle coefficient of variation of 0.41% was obtained for the integrated measurement process, including automatic referencing and sample repositioning, indicating high repeatability across successive measurements. In the sample-free control measurements, the system background under the LED-on condition was 79.84 ± 0.38 a.u., with a cycle-to-cycle CV of 0.48%, whereas the dark baseline count under the LED-off condition remained at 0.8 a.u. The data acquisition architecture supported continuous acquisition at defined integration intervals, with no missing data observed during the validation tests. The CW-OSL and LM-OSL measurements obtained with BeO were consistent with the expected signal profiles under constant and linearly modulated stimulation, respectively, supporting the experimental performance of the platform under different OSL protocols. LuminaOSL may therefore serve as a flexible experimental platform for research involving radiotherapy and radiological dosimetry, including potential applications in smart healthcare engineering, as well as personal and environmental dosimetry, characterization of OSL dosimeters and luminescent materials, and the development and comparison of different stimulation and readout protocols.

## Figures and Tables

**Table 1:** Comparison of the LuminaOSL platform with selected research-oriented OSL readers reported in the literature in terms of technical architecture, measurement capabilities, and reported performance characteristics.

Study	Stimulation & Detection	System Architecture & Control	Measurement Modes/Evaluated Materials	Main Feature/Performance
[7]	535 nm LED; GG-420; DM016C PMT; U-340/DUG11 filtering	Automated 8-position system; Arduino Due; Python GUI	CW-OSL, LM-OSL; BeO, Al_2_O_3_:C, quartz	Open-source, automated and modifiable multi-sample research reader
[8]	470 nm blue + 850 nm IR LEDs; H11870-02-Y005 PMT; UV filtering	Portable reader; manual sample holders; embedded control; Helios software	CW-OSL, IRSL; loess, Al_2_O_3_:C, BeO, NaCl	Portable dating/dosimetry screening; MDD ≈ 88 µGy (Al_2_O_3_:C), ≈265 µGy (BeO)
[9]	470 nm LED; PMT photon counting	Automated 24-card badge reader	CW-OSL; α-Al_2_O_3_:C	Individual monitoring; MMD ≈ 26 µGy; reproducibility ≈ 1.12%
[10]	≈450 nm LED cluster; PMT detection	Laboratory single-detector prototype	CW-OSL, POSL; Al_2_O_3_:C	Commercial-reader intercomparison; CW/POSL capability
[11]	473/532 nm lasers; PDM03-9107-USB photon counter	Compact single-sample standalone system; micro-PC/touch control	CW-OSL; LiCaAlF_6_:Eu,Y	Medical/radiotherapy dosimetry; MMD = 7 µGy (1 s)
[12]	470 nm LEDs; H11870-01 PMT; U-340 filtering	Portable/semi-automatic; NI DAQ; LabVIEW/C#	TL, CW-OSL, LM-OSL, TaOSL; multiple materials	Retrospective/emergency dosimetry; detection limit ≈ 0.02 mGy
[13]	470 nm blue + 850 nm IR LEDs; 9125B PMT; UG-1	Single-sample system; MCU-based control; N_2_ atmosphere	TL, CW-OSL, LM-OSL, POSL; multiple phosphors	Phosphor and defect characterization; multi-mode capability
[14]	442/532 nm lasers; P10232 PMT; UG-1	Single-sample drawer; shutter-controlled PC acquisition	CW-OSL; α-Al_2_O_3_:C	Radiotherapy dosimetry; MMD = 136 µGy; reproducibility ±3%
[20]	455 nm blue LED; Hamamatsu photosensor module; UV-region detection; optical filtering	Dedicated BeOmax reader; electronic control and software-based dose evaluation; coded encapsulated dosimeters	OSL; BeO (Thermalox 995™)	TU Dresden BeO dosimetry system; linear response from 10 µGy to 10 Gy; CV < 2% above 0.1 mGy
[39]	150 mW Ar + laser; LED-based modular stimulation	Modular research system; PC and microcontroller control	TL, CW-OSL, POSL; Al_2_O_3_:C, BeO, quartz, electronic components	Modular multi-material OSL/TL platform; configurable measurement capability
[40]	Laser-scanning optical stimulation and OSL detection	Prototype 2D laser-scanning reader with image reconstruction	OSL; Al_2_O_3_:C and Al_2_O_3_:C,Mg films	2D OSL dosimetry and spatial dose mapping
This Study	475 nm LED; GG-420/dichroic/U340; H7173A PMT	Automated 8-position system; auto-referencing; Arduino Due; RAM-buffered acquisition; Python GUI	CW-OSL, LM-OSL; BeO	Modular research platform; cycle-to-cycle CV = 0.41%; background CV = 0.48%; RAM-buffered data acquisition with complete data transfer

## Data Availability

No new data were created or analyzed in this study. Data sharing is not applicable to this article.

## References

[1] Murray A., Arnold L.J., Buylaert J.P., Guérin G., Qin J., Singhvi A.K., Smedley R., Thomsen K.J. (2021). Optically Stimulated Luminescence Dating Using Quartz. Nat. Rev. Methods Primers.

[2] Bobić M., Christensen J.B., Lee H., Choulilitsa E., Czerska K., Togno M., Safai S., Yukihara E.G., Winey B.A., Lomax A.J. (2024). Optically Stimulated Luminescence Dosimeters for Simultaneous Measurement of Point Dose and Dose-Weighted LET in an Adaptive Proton Therapy Workflow. Front. Oncol..

[3] Alghamdi H.M.S., Sanderson D.C.W., Cresswell A.J., Fitzgerald S. (2024). Radiological or Nuclear Emergency OSL Dosimetry Using Commonplace Salt. Radiat. Meas..

[4] Yukihara E.G., McKeever S.W.S. (2011). Optically Stimulated Luminescence: Fundamentals and Applications.

[5] Zhang J., Xiang Y., Wang C., Chen Y., Tjin S.C., Wei L. (2022). Recent Advances in Optical Fiber Enabled Radiation Sensors. Sensors.

[6] Bøtter-Jensen L., Andersen C.E., Duller G.A.T., Murray A.S. (2003). Developments in Radiation, Stimulation and Observation Facilities in Luminescence Measurements. Radiat. Meas..

[7] Maraba D., Bulur E. (2017). Design and Construction of an Automated OSL Reader with Open Source Software and Hardware. Radiat. Meas..

[8] Majgier R., Maternicki K., Mandowski A., Moska P., Biernacka M., Kreutzer S. (2025). The Helios OSL Reader: A Portable System for Dating and Dosimetry Applications. Geochronometria.

[9] Mishra D.R., Paliwal L., Sutar S.S., Singh A.K. (2020). Development of Optically Stimulated Luminescence Badge Reader System for Individual Monitoring of Radiation Workers. Radiat. Prot. Dosim..

[10] De Lazzari R., Souza E.H., Zanetti S.Y., Viana E.R., Malthez A.L.M.C. (2023). Intercomparison Study of Sensitivities from Commercial OSL Readers and a Newly Developed OSL Reader. Radiat. Prot. Dosim..

[11] Soni A., Khanderao R.N., Kakade N., Mishra D.R., Sharma S.D., Sapra B.K. (2025). A New Highly Sensitive Compact Laser Based OSL Reader—Application in Medical Dosimetry. Nucl. Instrum. Methods Phys. Res. Sect. B Beam Interact. Mater. At..

[12] Kim H., Park C.Y., Kim S.I., Kim M.C., Lee J. (2024). Development of a Prototype TL/OSL Reader for on-Site Use in a Large-Scale Radiological Accident. Nucl. Eng. Technol..

[13] Mishra D.R., Pal S., Bhowmick S., Paliwal L., Singh A.K., Sutar S.S., Bakshi A.K., Kanjilal A. (2021). Development of Wavelength-Resolved 3D-Integrated TL and OSL Reader System. Eng. Res. Express.

[14] Soni A., Agarwalla S.K., Kakade N.R., Mishra D.R., Sharma S.D. (2022). Development of Laser Based OSL Reader for Its Potential Application in Radiation Dosimetry. Nucl. Instrum. Methods Phys. Res. Sect. B Beam Interact. Mater. At..

[15] Bulur E. (1996). An Alternative Technique for Optically Stimulated Luminescence (OSL) Experiment. Radiat. Meas..

[16] Bulur E., Bøtter-Jensen L., Murray A.S. (2001). LM-OSL Signals from Some Insulators: An Analysis of the Dependency of the Detrapping Probability on Stimulation Light Intensity. Radiat. Meas..

[17] Bulur E., Göksu H.Y. (1998). OSL from BeO Ceramics: New Observations from an Old Material. Radiat. Meas..

[18] Jahn A., Sommer M., Liebmann M., Henniger J. (2011). Progress in 2D-OSL-Dosimetry with Beryllium Oxide. Radiat. Meas..

[19] Sommer M., Jahn A., Henniger J. (2011). A New Personal Dosimetry System for HP(10) and HP(0.07) Photon Dose Based on OSL-Dosimetry of Beryllium Oxide. Radiat. Meas..

[20] Jahn A., Sommer M., Ullrich W., Wickert M., Henniger J. (2013). The BeOmax System—Dosimetry Using OSL of BeO for Several Applications. Radiat. Meas..

[21] Sommer M., Henniger J. (2006). Investigation of a BeO-Based Optically Stimulated Luminescence Dosemeter. Radiat. Prot. Dosim..

[22] Kry S.F., Alvarez P., Cygler J.E., DeWerd L.A., Howell R.M., Meeks S., O’Daniel J., Reft C., Sawakuchi G., Yukihara E.G. (2020). AAPM TG 191: Clinical Use of Luminescent Dosimeters: TLDs and OSLDs. Med. Phys..

[23] Altunal V., Yegingil Z., Tuken T., Depci T., Ozdemir A., Guckan V., Nur N., Kurt K., Bulur E. (2018). Optically Stimulated Luminescence Characteristics of BeO Nanoparticles Synthesized by Sol-Gel Method. Radiat. Meas..

[24] Bossin L., Dal Bello R., Christensen J.B., Schischke S., Motta S., Togno M., Yukihara E.G. (2024). Performance of a BeO-Based Dosimetry System for Proton and Electron Beam Dose Measurements. Radiat. Meas..

[25] Kara E., Hicsonmez A. (2022). Physical and Dosimetric Characteristic Properties of BeO OSL for Clinical Dosimetric Measurements. Appl. Radiat. Isot..

[26] Kowalski J.P., Erickson B.G., Wu Q., Li X., Yoo S. (2025). Characterization, Commissioning, and Clinical Evaluation of a Commercial BeO Optically Stimulated Luminescence (OSL) System. J. Appl. Clin. Med. Phys..

[27] Han M.J., Kang S.W., Cho W., Kim J.S., Kim I.A., Chung J.B. (2024). Evaluation of Photoconductor and Scintillator Hybrid Dosimeters for Radiation Therapy Quality Assurance. J. Instrum..

[28] Lee M.S., Hwang D., Kim J.H., Lee J.S. (2019). Deep-Dose: A Voxel Dose Estimation Method Using Deep Convolutional Neural Network for Personalized Internal Dosimetry. Sci. Rep..

[29] Bulur E., Yeltik A. (2010). Optically Stimulated Luminescence from BeO Ceramics: An LM-OSL Study. Radiat. Meas..

[30] Yukihara E.G. (2020). A Review on the OSL of BeO in Light of Recent Discoveries: The Missing Piece of the Puzzle?. Radiat. Meas..

[31] Davis H., Siebers J.V., Wijesooriya K., Mistro M. (2025). Clinical Validation of MyOSLchip: A Beryllium Oxide Optically Stimulated Luminescent Dosimeter (OSLD) System in Radiotherapy Dosimetry. J. Appl. Clin. Med. Phys..

[32] Yadav P.J. (2021). Development of LED Based OSL Dosimeters for Personal Monitoring. J. Phys. Conf. Ser..

[33] Lukas E., Santos A.M.C., Ganija M., Veitch P. (2025). Evaluating the Effects of Increased Stimulation Power on a Beryllium Oxide Real-Time Optically Stimulated Luminescence Fibre-Coupled Dosimeter. Radiat. Meas..

[34] Photomultiplier Tube R3550A|Hamamatsu Photonics. https://www.hamamatsu.com/us/en/product/optical-sensors/pmt/pmt_tube-alone/head-on-type/R3550A.html.

[35] Bulur E. (2014). More on the TR-OSL Signal from BeO Ceramics. Radiat. Meas..

[36] Çam E., Çelik R., Sapmaz İ., Hoca S., İçhedef M., Karalı E.E., Kamer E.S., Ateş M., Biber Müftüler F.Z. (2026). Chronic 137Cs and 90Sr/90Y Radiotolerance and 99^m^Tc Uptake Capacity of Radon-Active Groundwater Bacteria: A Preliminary Assessment of Traits Relevant to Bioremediation. J. Environ. Radioact..

[37] Kara E., Woda C. (2023). Further Characterization of BeO Detectors for Applications in External and Medical Dosimetry. Radiat. Meas..

[38] Broadhead B., Noble C., Ramachandran P. (2022). A Direct Comparison of the Optically Stimulated Luminescent Properties of BeO and Al2O3 for Clinical In-Vivo Dosimetry. Phys. Eng. Sci. Med..

[39] Genicot J.L., Moyaerts M., Schroeyers W. (2011). Description of a Portable Devices Developed at SCK•CEN for OSL and TL Dosimetry. Radiat. Meas..

[40] Ahmed M.F., Shrestha N., Schnell E., Ahmad S., Akselrod M.S., Yukihara E.G. (2016). Characterization of Al2O3 Optically Stimulated Luminescence Films for 2D Dosimetry Using a 6 MV Photon Beam. Phys. Med. Biol..

[41] Polymeris G.S., Çoskun S., Tsoutsoumanos E., Konstantinidis P., Aşlar E., Şahiner E., Meriç N., Kitis G. (2021). Dose Response Features of Quenched and Reconstructed, TL and Deconvolved OSL Signals in BeO. Results Phys..

[42] Madden L., Lukas E., Santos A., Ganija M., Veitch P., Rosenfeld A., Li E. (2021). Deconvolution Analysis Improves Real-Time OSL of BeO Ceramic. Radiat. Meas..

[43] Kara E., Woda C. (2024). Correlation between Thermoluminescence and Optically Stimulated Luminescence Signal in BeO. Radiat. Meas..

[44] Abusaid W., Altunal V., Akdeniz Y., Guckan V., Ceyran G., Khandaker M.U., Yegingil Z. (2023). Studies of OSL Properties of Alkali- and Rare Earth-Doped BeO Based Novel Dosimeters for Applications in External Beam Radiotherapy. Radiat. Phys. Chem..

[45] Altunal V., Guckan V., Ozdemir A., Zydhachevskyy Y., Lawrence Y., Yu Y., Yegingil Z. (2022). Three Newly Developed BeO-Based OSL Dosimeters. J. Lumin..

[46] Altunal V., Jain M., Hayat S., Guckan V., Yegingil Z. (2022). Development of BeO:Na,Yb,Dy Ceramics for Optically Stimulated Luminescence Dosimetry. Radiat. Meas..

